# A Curious Pathological Phenomenon

**Published:** 1887-07

**Authors:** 


### A CURIOUS PATHOLOGICAL PHENOMENON.

By Dr. B. L. C.

A remarkable case has lately been communicated to the French Academy of Medicine.

A girl of fourteen to fifteen years of age swallowed a paper of steel needles seven years ago, from which no serious consequences appeared, but a few weeks since, while Miss N———, in full health and cheerful spirits, was embracing her mother, and fondly pressing her to her bosom, she caused her to exclaim : "Oh ! dear, you prick me," and lo ! behold, when Miss N—— released her mother, a needle was discovered protruding from under her finger nail. It came out shortly and entirely, followed, at brief intervals, by many other needles, making up almost the entire contents of the package she had swallowed seven years before, and without experiencing any inconvenience or pain. The most remarkable fact in this phenomenon is that the needles were not even oxidized. They have been sent to the Academy of Sciences.—*Translated from the Courrier Des Etats-Unis.*

